# Effects of surgery and propofol-remifentanil total intravenous anesthesia on cerebrospinal fluid biomarkers of inflammation, Alzheimer’s disease, and neuronal injury in humans: a cohort study

**DOI:** 10.1186/s12974-017-0950-2

**Published:** 2017-09-29

**Authors:** Andreas Pikwer, Markus Castegren, Sijal Namdar, Kaj Blennow, Henrik Zetterberg, Niklas Mattsson

**Affiliations:** 1 Centre for Clinical Research Sörmland, Uppsala University, Uppsala, Sweden; 2 Perioperative medicine and intensive care (PMI), Karolinska University Hospital and Clintec, Karolinska Institute, Stockholm, Sweden; 3 Department of Anesthesia, Mälarsjukhuset, Eskilstuna, Sweden; 4 Department of Psychiatry and Neurochemistry, Institute of Neuroscience and Physiology, the Sahlgrenska Academy at the University of Gothenburg, Möndal, Sweden; 5 Clinical Neurochemistry Laboratory, Sahlgrenska University Hospital, Mölndal, Sweden; 6 Department of Molecular Neuroscience, UCL Institute of Neurology, Queen Square, London, UK; 7 Clinical Memory Research Unit, Faculty of Medicine, Lund University, Lund, Sweden; 8 Department of Neurology, Skåne University Hospital, Lund, Sweden

**Keywords:** Anesthesia, Biomarkers, Cerebrospinal fluid, Inflammation, Surgery

## Abstract

**Background:**

Surgery and anesthesia have been linked to postoperative cognitive disturbance and increased risk of Alzheimer’s disease. It is not clear by which mechanisms this increased risk for cognitive disease is mediated. Further, amyloid β production has been suggested to depend on the sleep-wake cycle and neuronal activity. The aim of the present study was to examine if cerebrospinal fluid (CSF) concentrations of a number of biomarkers for Alzheimer’s disease-related processes, including amyloid β, neuronal injury, and inflammation, changed over time during intravenous anesthesia in surgical patients.

**Methods:**

We included patients scheduled for hysterectomy via laparotomy during general anesthesia with intravenous propofol and remifentanil. CSF samples were obtained before, during, and after surgery (5 h after induction) and tested for 27 biomarkers. Changes over time were tested with linear mixed effects models.

**Results:**

A total of 22 patients, all females, were included. The mean age was 50 years (± 9 SD). The mean duration of the anesthesia was 145 min (± 40 SD).

Interleukin (IL)-6, IL-8, monocyte chemoattractant protein 1, and vascular endothelial growth factor A increased over time. IL-15 and IL-7 decreased slightly over time. Macrophage inflammatory protein 1β and placental growth factor also changed significantly. There were no significant effects on amyloid β (Aβ) or tau biomarkers.

**Conclusions:**

Surgery and general anesthesia with intravenous propofol and remifentanil induce, during and in the short term after the procedure, a neuroinflammatory response which is dominated by monocyte attractants, without biomarker signs of the effects on Alzheimer’s disease pathology or neuronal injury.

**Electronic supplementary material:**

The online version of this article (10.1186/s12974-017-0950-2) contains supplementary material, which is available to authorized users.

## Background

Surgery and anesthesia have been linked to postoperative cognitive disturbance and to increased risk of future incidence of Alzheimer’s disease (AD) [1]. It is not clear by which mechanism this possible increased risk for AD is mediated. AD is believed to be caused by the accumulation of aggregated amyloid β (Aβ) peptides and phosphorylated tau (P-tau) proteins in the brain, which is reflected by low cerebrospinal fluid (CSF) levels of Aβ42 and high levels of total tau (T-tau) and P-tau, respectively [2, 3]. AD is also characterized by other brain changes, including microglial activation, which is reflected by altered levels of inflammatory CSF biomarkers [4].

Both in vitro and animal studies indicate that anesthetics might affect the neurochemical pathways of AD [5], but few studies have tested this in humans, especially when using intravenous anesthetics. Volatile anesthetics increase Aβ levels and P-tau levels in vitro (cell models) [6], in vivo (animal models) [7, 8], and in human CSF [5, 9]. Further, Aβ production has been suggested to depend on the sleep-wake cycle [10] and to be regulated by neuronal activity [11]. Intravenous anesthetics have been linked to increased tau phosphorylation in vitro [12]. One recent small study found an increase in several biomarkers for inflammation (such as Interleukin (IL)-6 and IL-8) associated with intravenous anesthesia and surgery in humans [13].

The studies described above suggest that anesthetics and surgery may influence neurochemical pathways relevant for AD. However, it is not clear if the neuroinflammatory response after anesthesia and surgery is coupled with effects on neuronal injury, Aβ and tau pathology, or if these different pathways may be triggered independently. We therefore tested a large number of CSF biomarkers related to neuroinflammation, Aβ, tau, and neuronal injury in parallel in a longitudinal study of patients undergoing surgery and anesthesia.

## Methods

The aim of the present study was to analyze CSF analytes related to Aβ and tau metabolism, neuronal injury, and inflammation before, during, and after intravenous anesthesia in a patient clientele without known neurological disease or impairment, to determine which neurochemical pathways that are preferentially affected by surgery and intravenous anesthesia.

### Study population

We included 22 patients scheduled for hysterectomy via laparotomy. Exclusion criteria were pre-existing neurologic or psychiatric disease and any contraindications for spinal anesthesia (including aortic stenosis, coagulopathy, hypovolemia, elevated intracranial pressure, and local infection).

### Study protocol

All patients received routine premedication including 50 mg Diclofenac and 4 mg Betamethasone. Prior to anesthesia, a spinal catheter was placed and baseline samples of 5 ml CSF were obtained. The Intralong spinal catheter system (Pajunk, Geisingen, Germany) with 21 G Sprotte needle and 25 G spinal catheter was used. No bacterial filter was used when the samples were collected since tests prior to the study showed that such filters adsorb Aβ42 (mean concentration of Aβ42 before filtration was 411 pg/ml and after filtration 226 pg/ml, *n* = 2).

All patients were given general anesthesia with intravenous propofol and remifentanil infusion using Marsh target control infusion (TCI) algorithm. Target cerebral concentrations were 4 μg/ml for propofol and 8 ng/ml for remifentanil. Patients were monitored with continuous pulse oximetry, continuous electrocardiography, and non-invasive blood pressure every 5 min. The nurse anesthetist documented the saturation, pulse, and blood pressure every 5 min. Pulse surgical bleeding was estimated from the amount of blood in the surgical suction device and blood absorbed in the surgical compresses.

Subsequent sampling of 5 ml of CSF was performed during general anesthesia (about 2 h after induction) and when the patients were fully awake (about 5 h after induction). The catheter was removed after the third sampling.

### Biochemical analysis

CSF was sampled in polypropylene tubes, centrifuged and frozen at −70 °C until analyses. CSF neurofilament light chain (NFL) was measured using the NF-light® ELISA kit (UmanDiagnostics AB, Umeå, Sweden). CSF T-tau and P-tau181 were analyzed using the INNOTEST hTAU Ag and PHOSPHO-TAU (181p) ELISA methods (Fujirebio Europe, Ghent, Belgium). CSF neurogranin was measured using an in-house electrochemiluminescence immunoassay on the MSD (Meso Scale Discovery system; Rockville, MD, USA) platform, as described previously [14]. CSF YKL-40 was determined by the Human chitinase-3 quantikine ELISA kit (R&D systems, Inc., Minneapolis, MN, USA). CSF Aβ38, Aβ40, and Aβ42 were measured using V-plex Peptide Panel 1 Kits Aβ38, Aβ40, and Aβ42 (MSD) according to the manufacturer’s protocol. CSF soluble amyloid precursor protein (sAPP)α and APPβ were measured using the multiplex soluble APP assay (MSD).

CSF fibroblast growth factor (FGF), placental growth factor (PlGF), Fms-related tyrosine kinase (Flt)1, and vascular endothelial growth factor (VEGF) D were measured using the Angiogenesis Panel 1 kit. CSF IP-10, monocyte chemoattractant protein 1 (MCP-1), and macrophage inflammatory protein (MIP-1β) were measured using the Chemokine Panel 1 kit. CSF IL-12/23p40, IL-15, IL-16, IL-7, and VEGF-A were measured using the Cytokine Panel 1 kit. CSF interferon gamma (IFN-γ), IL-6, and IL-8 were measured using the Proinflammatory Panel 1 kit. CSF C-reactive protein (CRP), serum amyloid A (SAA), soluble intercellular adhesion molecule-1 (sICAM-1), and soluble vascular cell adhesion molecule-1 (sVCAM-1) were measured using the Vascular Panel 2 kit. All these panels were from MSD. We excluded FGF and IFN-γ from the statistical analysis since the levels in most samples were below the detection limit.

All analyses were performed by board-certified laboratory technicians who were blinded to clinical information. All samples were analyzed according to protocols approved by the Swedish Board of Accreditation and Conformity Assessment, using single batches of reagents.

### Statistical analysis

Biomarker data and demographic data are presented as mean (± standard deviation (SD)). Changes over time were tested with linear mixed effects models with biomarkers as dependent variables (scaled and standardized to z-scores) and time (hours) as a predictor. The linear mixed effects models included random slopes and intercepts.

For each biomarker, we tested two linear mixed effects models; with or without restricted cubic splines to model time. Without splines, time was modeled with one parameter (β) for a linear relationship between time and the tested biomarker. With splines, time was modeled with two parameters (β1 and β2). This allowed for a non-linear relationship between time and the tested biomarker. The spline models used three knots, placed (by standard convention) at the 10th percentile, 50th percentile, and 90th percentile of the time scale. Note that with restricted cubic splines, the function is constrained to be linear in the tails (before the first and after the last knot). This is an advantage, since standard cubic splines may behave poorly beyond the knots.

For each biomarker, we calculated the Akaike information criterion (AIC) for the two competing models (with and without splines) to select between the models. A lower AIC represents a better fit of a statistical model, with a difference in AIC (ΔAIC) of > 2 representing some evidence, and > 10 representing very strong evidence, for differences between models (favoring the smaller AIC). If the spline model reduced the AIC by > 2 units, we therefore selected the spline model; otherwise, we selected the basic model [15].

*P* values were corrected for multiple comparisons with false discovery rate (FDR). Statistical analysis was done with R (R foundation for statistical computation, Vienna, Austria, 2016).

## Results

A total of 22 patients, all females, were included. The mean age was 50 years (± 9 SD). The mean duration of anesthesia was 145 min (± 40 SD). The three CSF samples were collected at 0 min, 131 ± 44 min, and 312 ± 51 min. The patients had preoperatively the mean lowest recorded oxygen saturation of 97 ± 5%, mean lowest recorded pulse of 55 ± 19 beats per minute, a mean lowest recorded systolic blood pressure of 81 ± 27 mmHg, and a mean surgical bleeding of 146 ± 137 ml. There were no neurological complications to the use of the spinal catheter. Catheter malfunction resulted in incomplete sampling in three patients (two patients lacked the second sample and one patient lacked the second and the third samples).

The levels of P-tau were below the detection limit in six samples (from three patients). The levels of neurogranin were below the detection limit in 14 samples (from six patients). The level of VEGF-D was below the detection limit in one sample. The biomarker levels are shown in Additional file 1: Table S1.

**Fig. 1:**
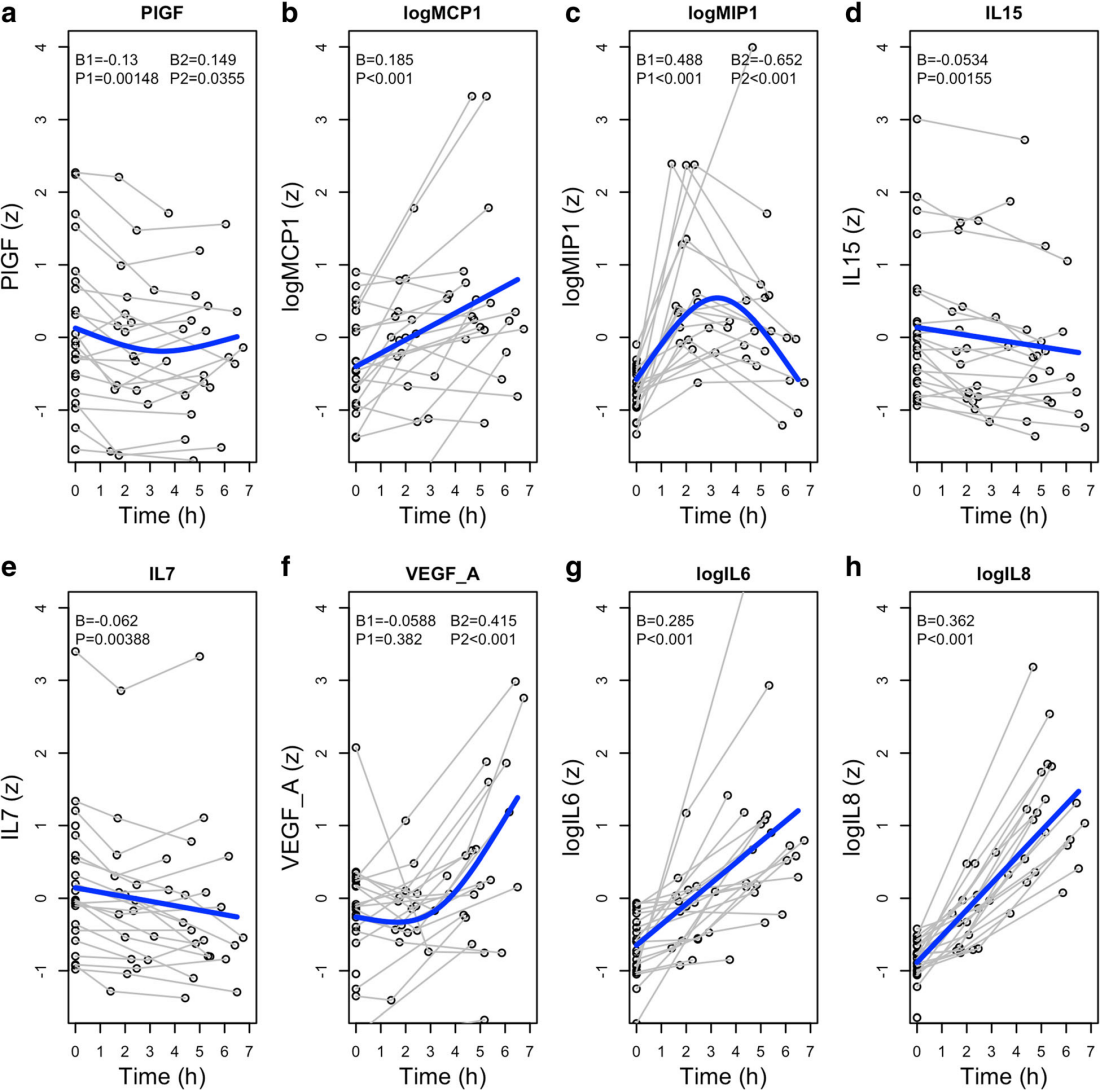
Dynamic changes in significant biomarkers. Biomarkers that had significant dynamic changes over time, when adjusted for multiple comparisons (see also Table 1 and Additional file 1: Table S1). **a**–**h** Each panel shows data for all individual subjects, and the average effect from a linear mixed effects model. For PIGF, MIP-1, and VEGF-A, a comparison of models favored a spline function to model time. For these biomarkers, β1 (linear parameter) and β2 (spline parameter) coefficients are presented. For the other biomarkers, time was modeled with a single parameter. *P* values are corrected for multiple comparisons (see Table 1 for uncorrected data)

Additional file 1: Table S1 summarizes effects for all 27 tested CSF biomarkers (for each biomarker, models with or without splines for time were compared and AICs were used for model selection, as explained in the methods). Eleven biomarkers had significant differences in trajectories over time (*P* < 0.05), and eight of these remained significant after FDR-correction for multiple comparisons (Table 1 and Fig. 1).

**Table 1:** Biomarkers over time

	Basic model				Spline model							ΔAIC favors spline
Biomarker	β	P	P (FDR)	AIC_basic_	β1	P1	P1 (FDR)	β2	P2	P2 (FDR)	AIC_spline_	
PlGF	−0.0488	0.0091	0.0320	112.0	*−0.13*	*0.0002*	*0.0015*	*0.149*	*0.0039*	*0.0355*	*109.7*	True
Log(MCP1)	*0.185*	*0.0001*	*0.0009*	*142.1*	0.18	0.0029	0.0125	0.00889	0.9090	0.9270	147.4	False
Log(MIP1)	0.151	0.0095	0.0320	176.2	*0.488*	*< 0.0001*	*< 0.0001*	*−0.652*	*< 0.0001*	*0.0002*	*160.1*	True
IL15	*−0.0534*	*0.0002*	*0.0015*	*95.2*	−0.0875	0.0017	0.0092	0.0641	0.1330	0.2565	99.5	False
IL-7	*−0.062*	*0.0007*	*0.0039*	*100.8*	−0.0899	0.0032	0.0125	0.055	0.2300	0.3653	105.7	False
VEGF-A	0.146	0.0045	0.0203	157.9	−0.0588	0.3110	0.3817	*0.415*	*< 0.0001*	*< 0.0001*	*143.2*	True
Log(IL-6)	*0.285*	*< 0.0001*	*0.0005*	*117.8*	0.265	0.0003	0.0022	0.0418	0.4850	0.6236	123.1	False
Log(IL-8)	*0.362*	*< 0.0001*	*< 0.0001*	*76.3*	0.312	0.0000	0.0000	0.102	0.0254	0.0686	77.9	False

Data is from linear mixed effects models for the eight biomarkers that changed over time, after correction for multiple comparisons (see Additional file 1: Table S1 for data on all biomarkers). Biomarkers were used as dependent variables (scaled and standardized to z-scores) and time (hours) was used as predictor. For each biomarker, we tested two models, with or without restricted cubic splines (using three knots) to model time. Without splines, time is modeled with one parameter (β), and with splines, times is modeled with two parameters (β1 and β2). For each biomarker, we calculated the Akaike information criterion (AIC) for the two models. AIC may be used to compare model fits, where a lower AIC is preferable and penalizes models with additional predictors (and thereby protects against overfitting). For biomarkers with AIC_basic_-AIC_spline_ < 2, we selected the basic model; otherwise we selected the spline model (selected model indicated with green shading). Data where *p* values are significant after correction for multiple comparisons [P (FDR)] are shown in italics. For example, for MIP1, the AIC selected the spline model, and both β1 (the linear component) and β2 (the cubic component) were significant, suggesting that MIP1 increased significantly during the first part of the study, and then decreased significantly during the second part of the study. In contrast, for IL-8, the AIC selected the non-spline model, and β was significant, suggesting that IL-8 increased continuously during the entire study duration. See Fig. 1 for visualizations of the significant effects

Different trajectories were identified among the biomarkers. The cytokines IL-6 and IL-8 and the chemokine MCP1 had clear monotone increases over time. VEGF-A also increased, but after an initial delay. MIP1β had a unique trajectory with an inverted u-shape with a return to baseline in the third sample. IL-15 and IL-7 decreased slightly over time. PlGF decreased slightly and stabilized or increased at the last sample. There were no significant effects on Aβ, tau, or neuronal injury biomarkers.

In a post-hoc analysis, we tested if baseline levels of T-tau, P-tau, or Aβ42 (as potential markers for amyloidopathy or tauopathy) were related to change in biomarkers, but we found no such effects that were significant after correction for multiple comparisons.

## Discussion

In this study on CSF biomarkers representing Aβ metabolism, tau metabolism, neuronal injury, and inflammatory pathways, we observed dynamic changes over time after intravenous anesthesia and surgery in specific inflammatory biomarkers. IL-8 had the most pronounced effect, which resonates well with earlier findings in an open heart, as well as in orthopedic, surgery [16–18]. Other biomarkers with significant effects over time were PlGF, MCP1, MIP-1β, IL-15, IL-7, VEGF-A, and IL-6. There were no effects on Aβ, tau, or neuronal injury biomarkers. Taken together, our findings show that intravenous anesthesia and surgery have effects on CNS inflammation, but we could not show effects on neuronal injury biomarkers or biomarkers reflecting the core AD pathologies Aβ and tau.

The fact that there were no effects on Aβ or tau biomarkers indicates that anesthesia and surgery do not have direct effects on Aβ or tau metabolism in the immediate postoperative period. Further, the lack of change in CSF Aβ levels during anesthesia and after waking up does not support that the overall Aβ release is strongly regulated by neuronal activity. The results are slightly in contrast with a study of intracortical brain surgery, which found that the levels of tau in CSF increased in samples analyzed at 10 and 24 h after induction [19], but it is possible that the surgery in that study contributed to the change in levels of CSF tau.

Several of the identified biomarkers have been described as proinflammatory, including IL-6, IL-8, and MCP-1, which all showed similar monotone increases over time. IL-6 is a potent proinflammatory cytokine that activates microglia and astrocytes and that has been described to be increased in CSF in AD [20, 21]. IL-8 has chemotactic functions for endothelial cells, T cells, neutrophils, and basophils [22] and monocytes [23]. Increased CSF levels of IL-8 have been shown to be associated with delirium in humans [24], but levels are not consistently increased in AD [25]. Both CSF IL-6 and IL-8 concentrations increased during an open cardiac surgery with 3–12-fold higher concentrations 24 h after the surgery compared with pre-surgery concentrations [16, 17], and similar changes were seen following orthopedic surgery [18]. MCP-1 (also called CCL2) is a potent chemotactic factor for monocytes and is increased in the neurons in patients with AD, especially in the hippocampal, the temporal, and the frontal areas [26]. Studies on CSF MCP-1 have shown either increased [27] or tendencies of increased levels in AD [28]. VEGF-A also increased in our patients but after an initial delay. VEGF-A is also a proinflammatory cytokine, which promotes cell migration and increased vascular permeability. AD patients have been shown to have increased CSF VEGF-A compared to healthy controls [29, 30]. In a rat model, increased VEGF was associated with hippocampal neurogenesis and improved cognition [31].

The observed rise in MCP-1, IL-6, and IL-8 associated with surgery and anesthesia confirms earlier experimental [32] and clinical findings [5, 13]. However, previous studies regarding surgery and anesthesia and neuroinflammation have mainly been conducted with volatile anesthetics. One recent animal study suggests that surgery and anesthesia is associated with neuroinflammation regardless of the type of anesthetics [33]. Hirsch et al. used unreported (but probably low) dose propofol infusion in combination with spinal anesthesia during surgery [13]. Although their study was much smaller (*N* = 10) than ours, they found similar results to that of ours that MCP-1, IL-6, and IL-8 increased during and after surgery. This may mean that it is primarily the surgery that induces the neuroinflammatory reaction, or that even a low-dose general anesthetic induces neuroinflammation.

Other biomarkers showed different trajectories over time. Among these, PlGF showed an initial decrease and a later stabilization. PlGF has a similar structure to VEGF-A and has also been known to increase vascular permeability and activate and recruit inflammatory cells [34]. It has been associated with ischemic brain injury, but to our knowledge not to AD [35]. Interestingly, in the abovementioned study on neurogenesis in rats, PlGF had a negative effect on neurogenesis [31].

MIP-1β (also called CCL4) had a unique trajectory with an initial increase and a later decrease during the study. MIP-1β is involved in both acute and chronic inflammation. It has mainly chemokinetic activity regarding T cells, monocytes, dendritic cells, and NK cells [36], which has mainly been studied in HIV [37]. There may be an association between MIP-1β and AD as MIP-1β could induce gathering of astrocytes and microglia in senile plaques [38]. Interestingly, in both the present study and a study of Hirsch et al. [13], the levels of MIP-1β increased rapidly and returned within hours to baseline levels. One could speculate that this is due to the natural kinetics of MIP-1β after inflammatory stimuli.

Finally, we observed a significant decrease in CSF levels of IL-7 and IL-15 over time. Bastian et al. reported the same pattern in postoperative systemic levels of IL-7 and IL-15 [39], although other studies have found them to be increased more than 24 h after the induction of inflammation [40, 41]. Both IL-7 and IL-15 affect the survival and memory cell formation of CD8+ T cells [42], and both have been linked to AD, although their function in AD remains unclear [43, 44].

One limitation of this study is the lack of concurrent blood samples. Previous studies have shown poor or no correlations between plasma and CSF concentrations of most inflammatory biomarkers in the perioperative period [13, 18]. The perioperative period is characterized by rapid changes in inflammatory activity, both in CNS and systemically. This might explain the poor correlation of plasma and CSF concentrations of inflammatory markers in this very specific situation. However, concurrent plasma samples would have facilitated the interpretation of our results.

Another limitation is that we cannot distinguish the effect of surgery from the effect of anesthesia. This is difficult to overcome since ethical concerns make it challenging to design studies that differentiate between effects of anesthesia and surgery. There is also a possibility that the indwelling spinal catheter may have inflammatory effects. To our knowledge, there is no study that compares indwelling spinal catheters and repeated lumbar punctures in this aspect.

Future studies may test if patients with significant inflammatory response are at increased risk for future cognitive decline and AD. Although we found significant effects on several inflammatory changes, the use of premedication with anti-inflammatory drugs might result in an underestimation of the proinflammatory changes. We included a wider array of biomarkers than other similar studies, but we still acknowledge that there may be other biomarkers that were not tested here and that may be important in AD, including for example IL-1 [45]. The fact that the last CSF sample was obtained only 5 h after induction opens for the possibility that there was no enough time for some biomarkers (such as Aβ and tau) to change significantly. However, effective β-secretase (BACE1) inhibition results in highly significant changes in CSP levels of Aβ within 3 h of administration [46]. This suggests that if intravenous anesthesia and surgery would have a direct effect on Aβ metabolism, we would have observed Aβ level changes in our CSF samples. Even so, there is a possibility that some CSF biomarkers may only change after the studied time period. The study was restricted to females, which was a consequence of the surgical context of hysterectomy. AD is more common in women than in men [47, 48], and sex-dependent changes in the immune system has been implicated in the pathogenesis of AD [49]. However, several of our main results were similar to those of the study of Hirsch et al. [13], which included seven men and three females. We therefore find it unlikely that our results were significantly biased by the restriction to female participants. Finally, it is important to note that the study population had a low risk of AD pathology since the patients were relatively young, had no symptoms of any neurologic decease, and most of them had normal baseline levels of Aβ and tau. However, we believe that this age group is relevant to study, since the first signs of AD pathology and cognitive decline may occur from around 50–60 years of age [50, 51].

## Conclusions

In summary, despite the premedication with a non-steroidal anti-inflammatory drug and a corticosteroid, we observed biomarker signs of acute neuroinflammation in this population of patients undergoing surgery and general anesthesia. In general, we were able to reproduce, in younger patients, the neuroinflammatory pattern seen by Hirsch et al. in their study with low-dose propofol infusion in combination with spinal anesthesia during orthopedic surgery; although, they tested a partly different set of inflammatory markers. A main finding is that the neuroinflammatory response was dominated by IL-6, IL-8, and MCIP-1, which are potent attractors of monocytes. The reduction in IL-7 and IL-15, which are primarily involved in T cell proliferation and memory, may suggest that the inflammatory pathway does not involve T cells. Further studies need to be conducted to assess the specific cellular response in the neuroinflammation associated with anesthesia and surgery and to assess if these responses are associated with delirium, cognitive decline, and development of AD. Future studies may also include groups with and without general anesthesia to clarify the specific effect of anesthesia versus the effect of surgery on neuroinflammation.

## Additional file

Additional file 1: Table S1. Biomarker levels in all samples. Prior to anesthesia, a spinal catheter was placed and baseline samples of CSF were obtained. Subsequent sampling of CSF was performed once during general anesthesia and finally when the patients were fully awake. (DOCX 22 kb)

## Abbreviations

Aβ: Amyloid β
AD: Alzheimer’s disease
AIC: Akaike information criterion
BACE1: β-secretase 1
CRP: C-reactive protein
CSF: Cerebrospinal fluid
FGF: Fibroblast growth factor
Flt: Fms-related tyrosine kinase
IFN-γ: Interferon gamma
IL: Interleukin
MCP-1: Monocyte chemoattractant protein 1
MIP: Macrophage inflammatory protein
NFL: Neurofilament light chain
PlGF: Placental growth factor
P-tau: Phosphorylated tau
sAPP: Soluble amyloid precursor protein
sICAM-1: Soluble intercellular adhesion molecule-1
SSA: Serum amyloid A
sVCAM-1: Soluble vascular cell adhesion molecule-1
TCI: Target control infusion
T-tau: Total tau
VEGF: Vascular endothelial growth factor

## References

[1] Chen PL, Yang CW, Tseng YK, Sun WZ, Wang JL, Wang SJ, Oyang YJ, Fuh JL (2014). Risk of dementia after anaesthesia and surgery. Br J Psychiatry.

[2] Barage SH, Sonawane KD. Amyloid cascade hypothesis: pathogenesis and therapeutic strategies in Alzheimer’s disease. Neuropeptides. 2015; 10.1016/j.npep.2015.06.008 26149638

[3] Jiang J, Jiang H (2015). Effect of the inhaled anesthetics isoflurane, sevoflurane and desflurane on the neuropathogenesis of Alzheimer's disease (review). Mol Med Rep.

[4] Rosen C, Zetterberg H (2013). Cerebrospinal fluid biomarkers for pathological processes in Alzheimer’s disease. Curr Opin Psychiatry.

[5] Tang JX, Baranov D, Hammond M, Shaw LM, Eckenhoff MF, Eckenhoff RG (2011). Human Alzheimer and inflammation biomarkers after anesthesia and surgery. Anesthesiology.

[6] Dong Y, Wu X, Xu Z, Zhang Y, Xie Z (2012). Anesthetic isoflurane increases phosphorylated tau levels mediated by caspase activation and Abeta generation. PLoS One.

[7] Dong Y, Zhang G, Zhang B, Moir RD, Xia W, Marcantonio ER, Culley DJ, Crosby G, Tanzi RE, Xie Z (2009). The common inhalational anesthetic sevoflurane induces apoptosis and increases beta-amyloid protein levels. Arch Neurol.

[8] Tang JX, Mardini F, Caltagarone BM, Garrity ST, Li RQ, Bianchi SL, Gomes O, Laferla FM, Eckenhoff RG, Eckenhoff MF (2011). Anesthesia in presymptomatic Alzheimer's disease: a study using the triple-transgenic mouse model. Alzheimers Dement.

[9] Zhang B, Tian M, Zheng H, Zhen Y, Yue Y, Li T, Li S, Marcantonio ER, Xie Z (2013). Effects of anesthetic isoflurane and desflurane on human cerebrospinal fluid abeta and tau level. Anesthesiology.

[10] Lucey BP, Bateman RJ (2014). Amyloid-beta diurnal pattern: possible role of sleep in Alzheimer’s disease pathogenesis. Neurobiol Aging.

[11] Brody DL, Magnoni S, Schwetye KE, Spinner ML, Esparza TJ, Stocchetti N, Zipfel GJ, Holtzman DM (2008). Amyloid-beta dynamics correlate with neurological status in the injured human brain. Science.

[12] Whittington RA, Virag L, Marcouiller F, Papon MA, El Khoury NB, Julien C, Morin F, Emala CW, Planel E (2011). Propofol directly increases tau phosphorylation. PLoS One.

[13] Hirsch J, Vacas S, Terrando N, Yuan M, Sands LP, Kramer J, Bozic K, Maze MM, Leung JM (2016). Perioperative cerebrospinal fluid and plasma inflammatory markers after orthopedic surgery. J Neuroinflammation.

[14] Portelius E, Zetterberg H, Skillback T, Tornqvist U, Andreasson U, Trojanowski JQ, Weiner MW, Shaw LM, Mattsson N, Blennow K (2015). Cerebrospinal fluid neurogranin: relation to cognition and neurodegeneration in Alzheimer’s disease. Brain.

[15] Burnham KP, Anderson DR, Burnham KP (2002). Model selection and multimodel inference : a practical information-theoretic approach.

[16] Reinsfelt B, Ricksten SE, Zetterberg H, Blennow K, Freden-Lindqvist J, Westerlind A (2012). Cerebrospinal fluid markers of brain injury, inflammation, and blood-brain barrier dysfunction in cardiac surgery. Ann Thorac Surg.

[17] Reinsfelt B, Westerlind A, Blennow K, Zetterberg H, Ricksten SE (2013). Open-heart surgery increases cerebrospinal fluid levels of Alzheimer-associated amyloid beta. Acta Anaesthesiol Scand.

[18] Bromander S, Anckarsater R, Kristiansson M, Blennow K, Zetterberg H, Anckarsater H, Wass CE (2012). Changes in serum and cerebrospinal fluid cytokines in response to non-neurological surgery: an observational study. J Neuroinflammation.

[19] Berger M, Nadler JW, Friedman A, McDonagh DL, Bennett ER, Cooter M, Qi W, Laskowitz DT, Ponnusamy V, Newman MF (2016). The effect of propofol versus isoflurane anesthesia on human cerebrospinal fluid markers of Alzheimer’s disease: results of a randomized trial. J Alzheimers Dis.

[20] Brosseron F, Krauthausen M, Kummer M, Heneka MT (2014). Body fluid cytokine levels in mild cognitive impairment and Alzheimer’s disease: a comparative overview. Mol Neurobiol.

[21] Spooren A, Kolmus K, Laureys G, Clinckers R, De Keyser J, Haegeman G, Gerlo S (2011). Interleukin-6, a mental cytokine. Brain Res Rev.

[22] Baggiolini M, Dewald B, Moser B (1997). Human chemokines: an update. Annu Rev Immunol.

[23] Baggiolini M, Dewald B, Moser B (1994). Interleukin-8 and related chemotactic cytokines—CXC and CC chemokines. Adv Immunol.

[24] MacLullich AM, Edelshain BT, Hall RJ, de Vries A, Howie SE, Pearson A, Middleton SD, Gillies F, Armstrong IR, White TO (2011). Cerebrospinal fluid interleukin-8 levels are higher in people with hip fracture with perioperative delirium than in controls. J Am Geriatr Soc.

[25] Wennstrom M, Surova Y, Hall S, Nilsson C, Minthon L, Hansson O, Nielsen HM (2015). The inflammatory marker YKL-40 is elevated in cerebrospinal fluid from patients with Alzheimer’s but not Parkinson’s disease or dementia with Lewy bodies. PLoS One.

[26] Liao Y, Qi XL, Cao Y, Yu WF, Ravid R, Winblad B, Pei JJ, Guan ZZ: Elevations in the levels of NF-kappaB and inflammatory chemotactic factors in the brains with Alzheimer’s disease—one mechanism may involve alpha3 nicotinic acetylcholine receptor. Curr Alzheimer Res 2016. 10.2174/1567205013666160703174254 27396406

[27] Galimberti D, Schoonenboom N, Scarpini E, Scheltens P, Dutch-Italian Alzheimer Research G (2003). Chemokines in serum and cerebrospinal fluid of Alzheimer’s disease patients. Ann Neurol.

[28] Mattsson N, Tabatabaei S, Johansson P, Hansson O, Andreasson U, Mansson JE, Johansson JO, Olsson B, Wallin A, Svensson J (2011). Cerebrospinal fluid microglial markers in Alzheimer’s disease: elevated chitotriosidase activity but lack of diagnostic utility. NeuroMolecular Med.

[29] Del Bo R, Scarlato M, Ghezzi S, Martinelli Boneschi F, Fenoglio C, Galbiati S, Virgilio R, Galimberti D, Galimberti G, Crimi M (2005). Vascular endothelial growth factor gene variability is associated with increased risk for AD. Ann Neurol.

[30] Tarkowski E, Issa R, Sjogren M, Wallin A, Blennow K, Tarkowski A, Kumar P (2002). Increased intrathecal levels of the angiogenic factors VEGF and TGF-beta in Alzheimer’s disease and vascular dementia. Neurobiol Aging.

[31] During MJ, Cao L (2006). VEGF, a mediator of the effect of experience on hippocampal neurogenesis. Curr Alzheimer Res.

[32] Cibelli M, Fidalgo AR, Terrando N, Ma D, Monaco C, Feldmann M, Takata M, Lever IJ, Nanchahal J, Fanselow MS (2010). Role of interleukin-1beta in postoperative cognitive dysfunction. Ann Neurol.

[33] Zhang J, Tan H, Jiang W, Zuo Z (2015). The choice of general anesthetics may not affect neuroinflammation and impairment of learning and memory after surgery in elderly rats. J NeuroImmune Pharmacol.

[34] Tammela T, Enholm B, Alitalo K, Paavonen K (2005). The biology of vascular endothelial growth factors. Cardiovasc Res.

[35] Du H, Li P, Pan Y, Li W, Hou J, Chen H, Wang J, Tang H (2010). Vascular endothelial growth factor signaling implicated in neuroprotective effects of placental growth factor in an in vitro ischemic model. Brain Res.

[36] Maurer M, von Stebut E (2004). Macrophage inflammatory protein-1. Int J Biochem Cell Biol.

[37] Cocchi F, DeVico AL, Garzino-Demo A, Arya SK, Gallo RC, Lusso P (1995). Identification of RANTES, MIP-1 alpha, and MIP-1 beta as the major HIV-suppressive factors produced by CD8+ T cells. Science.

[38] Kauwe JS, Bailey MH, Ridge PG, Perry R, Wadsworth ME, Hoyt KL, Staley LA, Karch CM, Harari O, Cruchaga C (2014). Genome-wide association study of CSF levels of 59 alzheimer’s disease candidate proteins: significant associations with proteins involved in amyloid processing and inflammation. PLoS Genet.

[39] Bastian D, Tamburstuen MV, Lyngstadaas SP, Reikeras O (2008). Systemic and local cytokine kinetics after total hip replacement surgery. Eur Surg Res.

[40] Inoue S, Unsinger J, Davis CG, Muenzer JT, Ferguson TA, Chang K, Osborne DF, Clark AT, Coopersmith CM, McDunn JE (2010). IL-15 prevents apoptosis, reverses innate and adaptive immune dysfunction, and improves survival in sepsis. J Immunol.

[41] O'Sullivan ST, Lederer JA, Horgan AF, Chin DH, Mannick JA, Rodrick ML (1995). Major injury leads to predominance of the T helper-2 lymphocyte phenotype and diminished interleukin-12 production associated with decreased resistance to infection. Ann Surg.

[42] Rubinstein MP, Lind NA, Purton JF, Filippou P, Best JA, McGhee PA, Surh CD, Goldrath AW (2008). IL-7 and IL-15 differentially regulate CD8+ T-cell subsets during contraction of the immune response. Blood.

[43] Hall JR, Johnson LA, Barber RC, Vo HT, Winter AS, O'Bryant SE, Texas Alzheimer's R, Care C (2012). Biomarkers of basic activities of daily living in Alzheimer’s disease. J Alzheimers Dis.

[44] Rentzos M, Rombos A (2012). The role of IL-15 in central nervous system disorders. Acta Neurol Scand.

[45] Griffin WS, Liu L, Li Y, Mrak RE, Barger SW (2006). Interleukin-1 mediates Alzheimer and Lewy body pathologies. J Neuroinflammation.

[46] May PC, Dean RA, Lowe SL, Martenyi F, Sheehan SM, Boggs LN, Monk SA, Mathes BM, Mergott DJ, Watson BM (2011). Robust central reduction of amyloid-beta in humans with an orally available, non-peptidic beta-secretase inhibitor. J Neurosci.

[47] Qiu C, Kivipelto M, von Strauss E (2009). Epidemiology of Alzheimer’s disease: occurrence, determinants, and strategies toward intervention. Dialogues Clin Neurosci.

[48] Mielke MM, Vemuri P, Rocca WA (2014). Clinical epidemiology of Alzheimer’s disease: assessing sex and gender differences. Clin Epidemiol.

[49] Begum AN, Cunha C, Sidhu H, Alkam T, Scolnick J, Rosario ER, Ethell DW (2014). Women with the Alzheimer's risk marker ApoE4 lose Abeta-specific CD4(+) T cells 10-20 years before men. Transl Psychiatry.

[50] Jansen WJ, Ossenkoppele R, Knol DL, Tijms BM, Scheltens P, Verhey FR, Visser PJ (2015). Amyloid biomarker study G, Aalten P, Aarsland D et al: prevalence of cerebral amyloid pathology in persons without dementia: a meta-analysis. JAMA.

[51] Caselli RJ, Dueck AC, Osborne D, Sabbagh MN, Connor DJ, Ahern GL, Baxter LC, Rapcsak SZ, Shi J, Woodruff BK (2009). Longitudinal modeling of age-related memory decline and the APOE epsilon4 effect. N Engl J Med.

