# Supplementary material for: Effects of surgery and propofol-remifentanil total intravenous anesthesia on cerebrospinal fluid biomarkers of inflammation, Alzheimer’s disease, and neuronal injury in humans: a cohort study
Source: J Neuroinflammation. 2017 Sep 29;14:193. doi: 10.1186/s12974-017-0950-2 (PMC5622541; doi:10.1186/s12974-017-0950-2)
Supplement: Additional file 1: Table S1. — Biomarker levels in all samples. Prior to anesthesia, a spinal catheter was placed and baseline samples of CSF were obtained. Subsequent sampling of CSF was performed once during general anesthesia and finally when the patients were fully awake. (DOCX 22 kb) [file 12974_2017_950_MOESM1_ESM.docx]

|  | **Basic model** | | | | **Spline model** | | | | | | | **ΔAIC**  **favors spline** |
| --- | --- | --- | --- | --- | --- | --- | --- | --- | --- | --- | --- | --- |
| **Biomarker** | **β** | **P** | **P (FDR)** | **AIC**_basic_ | **β1** | **P1** | **P1 (FDR)** | **β2** | **P2** | **P2 (FDR)** | **AIC**_spline_ |  |
| **NFL** | -0,0389 | 0,1870 | 0,2805 | 137,0 | -0,0217 | 0,6500 | 0,7020 | -0,0339 | 0,6480 | 0,7607 | 142,2 | FALSE |
| **T-tau** | 0,0186 | 0,3180 | 0,4293 | 116,0 | -0,0392 | 0,2370 | 0,3267 | 0,113 | 0,0371 | 0,0835 | 118,0 | FALSE |
| **P-tau** | 0,00822 | 0,6640 | 0,7258 | 111,3 | -0,0546 | 0,0676 | 0,1217 | **0,125** | **0,0079** | **0,0426** | 111,0 | FALSE |
| **Neurogranin** | 0,0535 | 0,0830 | 0,1588 | 141,0 | 0,00322 | 0,9480 | 0,9480 | 0,1 | 0,1940 | 0,3274 | 144,7 | FALSE |
| **YKL-40** | -0,0196 | 0,0751 | 0,1560 | 69,7 | -0,041 | 0,0287 | 0,0554 | 0,0421 | 0,1490 | 0,2682 | 75,0 | FALSE |
| **Aβ38** | -0,0286 | 0,1830 | 0,2805 | 121,3 | **-0,0987** | **0,0064** | **0,0173** | **0,139** | **0,0133** | **0,0489** | 122,0 | FALSE |
| **Aβ40** | -0,0361 | 0,0882 | 0,1588 | 121,0 | **-0,106** | **0,0039** | **0,0133** | **0,137** | **0,0151** | **0,0489** | 121,9 | FALSE |
| **Aβ42** | -0,0269 | 0,2460 | 0,3496 | 129,5 | **-0,106** | **0,0098** | **0,0241** | **0,155** | **0,0163** | **0,0489** | 130,2 | FALSE |
| **sAPPα** | -0,0176 | 0,1540 | 0,2599 | 81,3 | **-0,0599** | **0,0046** | **0,0138** | **0,0828** | **0,0114** | **0,0489** | 82,4 | FALSE |
| **sAPPβ** | -0,00216 | 0,8620 | 0,8620 | 79,6 | -0,0439 | 0,0273 | 0,0554 | **0,0829** | **0,0076** | **0,0426** | 80,0 | FALSE |
| **PlGF** | **-0,0488** | **0,0091** | **0,0320** | 112,0 | **-0,13** | **0,0002** | **0,0015** | **0,149** | **0,0039** | **0,0355** | 109,7 | TRUE |
| **Flt** | 0,00541 | 0,6720 | 0,7258 | 92,6 | -0,0169 | 0,5000 | 0,5625 | 0,0436 | 0,2810 | 0,4215 | 98,2 | FALSE |
| **VEGF-D** | -0,0367 | 0,0420 | 0,1031 | 111,3 | **-0,0897** | **0,0118** | **0,0266** | 0,113 | 0,0582 | 0,1209 | 114,6 | FALSE |
| **Log(IP-10)** | 0,0129 | 0,7350 | 0,7633 | 137,9 | 0,00974 | 0,8480 | 0,8806 | 0,00685 | 0,9270 | 0,9270 | 143,3 | FALSE |
| **Log(MCP1)** | **0,185** | **0,0001** | **0,0009** | 142,1 | **0,18** | **0,0029** | **0,0125** | 0,00889 | 0,9090 | 0,9270 | 147,4 | FALSE |
| **Log(MIP1)** | **0,151** | **0,0095** | **0,0320** | 176,2 | **0,488** | **0,0000** | **0,0000** | **-0,652** | **0,0000** | **0,0002** | 160,1 | TRUE |
| **Log(IL-12/23p40)** | -0,00775 | 0,6250 | 0,7258 | 84,1 | -0,0193 | 0,4410 | 0,5177 | 0,0225 | 0,5460 | 0,6701 | 90,5 | FALSE |
| **IL15** | **-0,0534** | **0,0002** | **0,0015** | 95,2 | **-0,0875** | **0,0017** | **0,0092** | 0,0641 | 0,1330 | 0,2565 | 99,5 | FALSE |
| **Log(IL-16)** | 0,0357 | 0,5380 | 0,6603 | 161,8 | -0,0944 | 0,2570 | 0,3304 | 0,264 | 0,0345 | 0,0835 | 161,6 | FALSE |
| **IL-7** | **-0,062** | **0,0007** | **0,0039** | 100,8 | **-0,0899** | **0,0032** | **0,0125** | 0,055 | 0,2300 | 0,3653 | 105,7 | FALSE |
| **VEGF-A** | **0,146** | **0,0045** | **0,0203** | 157,9 | -0,0588 | 0,3110 | 0,3817 | **0,415** | **0,0000** | **0,0000** | 143,2 | TRUE |
| **Log(IL-6)** | **0,285** | **0,0000** | **0,0005** | 117,8 | **0,265** | **0,0003** | **0,0022** | 0,0418 | 0,4850 | 0,6236 | 123,1 | FALSE |
| **Log(IL-8)** | **0,362** | **0,0000** | **0,0000** | 76,3 | **0,312** | **0,0000** | **0,0000** | 0,102 | 0,0254 | 0,0686 | 77,9 | FALSE |
| **CRP** | -0,0382 | 0,0315 | 0,0851 | 100,1 | -0,0447 | 0,1270 | 0,2081 | 0,0128 | 0,7770 | 0,8741 | 106,4 | FALSE |
| **SAA** | -0,03 | 0,3590 | 0,4616 | 148,6 | -0,0636 | 0,2420 | 0,3267 | 0,0657 | 0,4350 | 0,6182 | 153,2 | FALSE |
| **sICAM-1** | -0,0656 | 0,0189 | 0,0567 | 141,9 | -0,0707 | 0,1910 | 0,2865 | 0,00967 | 0,9090 | 0,9270 | 147,0 | FALSE |
| **sVCAM-1** | -0,0338 | 0,0703 | 0,1560 | 119,0 | -0,0572 | 0,1310 | 0,2081 | 0,0438 | 0,4680 | 0,6236 | 124,3 | FALSE |

**Additional file 1: Table S1. Biomarkers over time**

Data is from linear mixed effects models with biomarkers as dependent variables (scaled and standardized to z-scores) and time (hours) as predictor. For each biomarker we tested two models; with or without restricted cubic splines (using 3 knots) to model time. Without splines, time is modelled with one parameter (β) and with splines, times is modelled with two parameters (β1 and β2). For each biomarker, we calculated the Akaike information criterion (AIC) for the two models. AIC may be used to compare model fits, where a lower AIC is preferable, and penalizes models with additional predictors (and thereby protects against overfitting). For biomarkers with AIC_basic_-AIC_spline_ < 2 we selected the basic model, otherwise we selected the spline model (selected model indicated with green shading). Data where p-values are significant after correction for multiple comparisons [P (FDR)] are shown in bold. The eight models that remained significant are shown in plots in Figure 1.
